# Targeting ApoC3 Paradoxically Aggravates Atherosclerosis in Hamsters With Severe Refractory Hypercholesterolemia

**DOI:** 10.3389/fcvm.2022.840358

**Published:** 2022-02-02

**Authors:** Yitong Xu, Jiabao Guo, Ling Zhang, Guolin Miao, Pingping Lai, Wenxi Zhang, Lili Liu, Xinlin Hou, Yuhui Wang, Wei Huang, George Liu, Mingming Gao, Xunde Xian

**Affiliations:** ^1^Laboratory of Lipid Metabolism, Institute of Basic Medicine, Hebei Medical University, Shijiazhuang, China; ^2^Institute of Cardiovascular Sciences and Key Laboratory of Molecular Cardiovascular Sciences, Ministry of Education, School of Basic Medical Sciences, Peking University, Beijing, China; ^3^Department of Pediatrics, Peking University First Hospital, Beijing, China; ^4^Beijing Key Laboratory of Cardiovascular Receptors Research, Peking University Third Hospital, Beijing, China

**Keywords:** ApoC3, atherosclerosis, LDLR, Syrian golden hamster, hypertriglyceridemia

## Abstract

**Rationale:**

ApoC3 plays a central role in the hydrolysis process of triglyceride (TG)-rich lipoproteins mediated by lipoprotein lipase (LPL), which levels are positively associated with the incidence of cardiovascular disease (CVD). Although targeting ApoC3 by antisense oligonucleotide (ASO), Volanesorsen markedly reduces plasma TG level and increase high-density lipoprotein cholesterol (HDL-C) in patients with hypertriglyceridemia (HTG), the cholesterol-lowering effect of ApoC3 inhibition and then the consequential outcome of atherosclerotic cardiovascular disease (ASCVD) have not been reported in patients of familial hypercholesterolemia (FH) with severe refractory hypercholesterolemia yet.

**Objective:**

To investigate the precise effects of depleting ApoC3 on refractory hypercholesterolemia and atherosclerosis, we crossed ApoC3-deficient hamsters with a background of LDLR deficiency to generate a double knockout (DKO) hamster model (LDLR^−/−^, XApoC3^−/−^, DKO).

**Approach and Results:**

On the standard laboratory diet, DKO hamsters had reduced levels of plasma TG and total cholesterol (TC) relative to LDLR^−/−^ hamsters. However, upon high-cholesterol/high-fat (HCHF) diet feeding for 12 weeks, ApoC3 deficiency reduced TG level only in female animals without affecting refractory cholesterol in the circulation, whereas apolipoprotein A1 (ApoA1) levels were significantly increased in DKO hamsters with both genders. Unexpectedly, loss of ApoC3 paradoxically accelerated diet-induced atherosclerotic development in female and male LDLR^−/−^ hamsters but ameliorated fatty liver in female animals. Further analysis of blood biological parameters revealed that lacking ApoC3 resulted in abnormal platelet (PLT) indices, which could potentially contribute to atherosclerosis in LDLR^−/−^ hamsters.

**Conclusions:**

In this study, our novel findings provide new insight into the application of ApoC3 inhibition for severe refractory hypercholesterolemia and ASCVD.

## Highlights

- ApoC3 deficiency reduces plasma triglyceride and total cholesterol levels in female and male LDLR^−/−^ hamsters on a standard laboratory diet.
- Loss of ApoC3 only lowers circulating triglyceride level in female LDLR^−/−^ hamsters without affecting severe refractory hypercholesterolemia after high-cholesterol/high-fat diet feeding.
- Targeting ApoC3 paradoxically exacerbates diet-induced atherosclerosis in LDLR^−/−^ hamsters independent of gender, but only protects against fatty liver in female animals.

## Introduction

Elevated triglyceride (TG) levels caused by genetic or environmental factors are positively associated with the increased incidence of atherosclerotic cardiovascular disease (ASCVD) and acute pancreatitis (AP) (1). Lipoprotein lipase (LPL) is a key rate-limiting enzyme that plays a central role in modulating TG metabolism. Loss-of-function mutations in the *LPL* gene cause severe hypertriglyceridemia (HTG) (2). To our knowledge, several important regulators of LPL enzymatic activity have been identified in the past decades, including activators such as apolipoprotein C-II (ApoC2) and apolipoprotein A-V (ApoA5), and inhibitors such as apolipoprotein C-III (ApoC3) and angiopoietin-like 3/4/8 (ANGPTL3/4/8). Although functional studies show that ApoC2 and ApoA5 are required for LPL-mediated TG hydrolysis process *in vitro* and *in vivo*, overexpression of human ApoC2 in mice unexpectedly elicits HTG and ApoA5-deficient mice have no obvious HTG under the condition of regular standard diet (3), implying that TG metabolism is complicated *in vivo* and HTG cannot be solved when only targeting activators involved in LPL activity.

Unlike the contradictory data from the LPL activator, abrogating the inhibitory effects of ApoC3 and ANGPTLs on LPL activity consistently reduces circulating TG levels, thus yielding promising outcomes of dyslipidemia in different experimental animal models (4). Recently, ANGPTL3 and ApoC3 have been reported to be potential therapeutic targets for the treatment of HTG in clinical trials. For example, evinacumab, an antibody that inhibits ANGPTL3 not only reduces TG level in circulation by enhancing LPL activity but also significantly decreases low-density lipoprotein cholesterol (LDL-C) in patients with familial hypercholesterolemia (FH) in two independent phase 3 trials (5, 6). However, although targeting ApoC3 by antisense oligonucleotide (ASO), volanesorsen, markedly reduces plasma TG level in patients with HTG (7, 8), the cholesterol-lowering effect of ApoC3 inhibition and then the consequential outcomes of atherosclerotic cardiovascular disease (ASCVD) have not been reported yet in patients with FH so far.

Previously, using CRISPR/Cas9 editing our group deleted the *Apoc3* gene from the Syrian golden hamster model that replicates human metabolic features, and we found that lack of ApoC3 significantly decreased plasma TG and total cholesterol (TC) levels, meanwhile increased high-density lipoprotein cholesterol (HDL-C) in hamsters fed with high-cholesterol/high-fat (HCHF) diet (9), suggesting that ApoC3 deficiency ameliorates diet-induced combined dyslipidemia. However, in agreement with the observations from the clinical trials, whether the beneficial effect of lacking ApoC3 relies on the presence of low-density lipoprotein receptor (LDLR) and whether ApoC3 inhibition can be applied for the treatment of severe refractory hypercholesterolemia and atherosclerosis due to LDLR deficiency still needs to be elucidated. In this study, we crossed ApoC3-deficient hamsters with a background of LDLR deficiency to generate a double knockout (DKO) hamster model (LDLR^−/−^ XApoC3^−/−^, DKO) and investigated the role of ApoC3 inhibition in lipid metabolism and atherosclerosis in the hamster model of FH. Our experimental evidence provides new insight into the possibility that ApoC3 will be a potential therapeutic target for the treatment of FH and atherosclerosis.

## Materials and Methods

### Animals

Golden Syrian hamsters were purchased from Vital River Laboratories (Beijing, China). Homozygous LDLR-deficient (LDLR^−/−^) hamsters and ApoC3^−/−^ hamsters were generated by CRISPR/ Cas9 genetic editing system in our laboratory as described previously (9, 10). In this study, ApoC3^−/−^ hamsters were crossed with LDLR^−/−^ hamsters to obtain homozygous double mutant animals (DKO), and LDLR^−/−^ hamsters were used as a control. The animals were maintained on a 14-h light/10-h dark cycle at 24°C and fed either a standard laboratory diet or HCHF diet (0.5% cholesterol and 20% fat) with water *ad libitum*. Both male and female animals were used in our study. All procedures were followed to the guidelines of Laboratory Animal Care (NIH Publication No. 85Y23, revised 1996), and the experimental protocol was approved by the Animal Care Committee, Peking University Health Science Center (LA2015-012).

### The Assays of Plasma Lipids and (Apo)Lipoproteins

Blood samples were collected from the retro-orbital plexus of the hamsters after 12-h fasting under isoflurane anesthesia. The plasma TC and TG levels were determined enzymatically using commercially available kits (Zhongsheng Beikong, Beijing) as described previously (9).

To analyze the lipid distribution, fast protein liquid chromatography (FPLC) of plasma lipoproteins was performed using 200 μl of pooled plasma samples from 6 animals with indicated genotypes, which were filtered by 0.22-mm filters and then applied to Tricorn high-performance Superose S-6 10/300GL columns (Amersham Biosciences), eluting with PBS at a constant flow rate of 0.25 ml/min. Eluted fractions (500 μl per fraction) were assessed for TG and cholesterol concentrations using the same TG and cholesterol kits as described above.

### Western Blots

The concentrations of apolipoprotein B (ApoB), apolipoprotein E (ApoE), and apolipoprotein A1 (ApoA1) in eluted fractions were detected by western blots. Briefly, every 3 consecutive fractions were equally pooled together, and 15 μl of pooled fractions was mixed with 4X SDS loading buffer (0.1 M Tris-HCl, 724 pH 6.8, 2% SDS, 5% β-mercaptoethanol, 10% glycerol, and 0.05% bromophenol blue). The mixtures were boiled at 95°C for 10 min. Proteins were separated by 4–20% SDS-PAGE and transferred to a nitrocellulose membrane for western blotting using different antibodies against ApoB, ApoE, and ApoA1. The following antibodies were used: ApoA1 (sc-30089, Santa Cruz Biotechnology, USA, rabbit polyclonal IgG, 1:1,000), ApoE (178479, Millipore, goat polyclonal IgG, 1:5,000), and ApoB (178467, Millipore, goat polyclonal IgG, 1:5,000).

### Pathological Analysis

To investigate atherosclerotic lesions and lipid accumulation in different tissues, all animals fed with HCHF diet for 16 weeks were perfused with cold (phosphate-buffered saline) PBS and then fixed by 4% paraformaldehyde PFA. The whole aorta, heart, and liver were harvested and embedded in OCT solution. A total of 10 μm of frozen cross-sectioned slices of aortic root and liver was used for morphological analysis. The atherosclerotic plaques in whole aortas (en-face) and cross-sectioned slices were visualized using 0.3% oil red O (ORO) solution (Sigma-Aldrich, St. Louis, MO, USA).

For immunofluorescence staining, the cluster of differentiation 68 (CD68) and ApoB in aortic root and liver were analyzed using primary antibodies against CD68 (1:100 rabbit polyclonal IgG; BA3638, BOSTER, California, USA) and ApoB antibody (1:100 goat polyclonal IgG; 178467, Millipore, Massachusetts, USA), respectively. The slices were then incubated with appropriate biotinylated second antibodies [1:100, Donkey anti-Goat IgG (H+L) Cross-Adsorbed Secondary Antibody, Alexa Fluor 633; Alexa Fluor 555; Thermo Fisher, Massachusetts, USA).

### Test of Blood Biochemical Parameters

A total of 20 μl of fresh blood samples was used for the measurement of the following biochemical parameters: the count platelet (PLT) count, mean platelet volume (MPV), platelet distribution width (PDW), and plateletcrit (PCT).

### Lecithin–Cholesterol Acyltransferase Activity Assays

Lecithin–cholesterol acyltransferase (LCAT) activity was performed using a commercial kit (Sigma, MAK107-1KT). The fluorometric substrates were incubated with 4 μl plasma from LDLR^−/−^ and DKO hamsters at 37°C for 2.5 h. The heat-inactivated plasma was used as a negative control to eliminate endogenous autofluorescence interference. The emission spectrum of the substrate reagent showed two distinct peaks at 390 and 470 nm, respectively. After hydrolysis of the substrate by LCAT, the fluorescence signal of 390/470 was increased. LCAT activity was evaluated by a change in the ratio of fluorescence intensity at 390 to 470 nm after excluding the value of endogenous autofluorescence.

## Statistical Analysis

All data were expressed as the mean ± SEM and evaluated using two-tailed Student's *t*-test for two groups with one variable tested and equal variances, one-way ANOVA with Dunnett's *post-hoc* or Tukey's *post-hoc* for multiple groups with only variable tested, or two-way ANOVA with Sidak's *post-hoc* for plaque quantification. The differences were considered to be significant at *p* < 0.05. The software used for data analysis was ImageJ (NIH) and Prism 8.0 (GraphPad Software).

## Results

### Effects of Depleting ApoC3 on Plasma Lipid Metabolism in LDLR^–/–^ Hamsters Under Different Dietary Conditions

**Figure 1:**
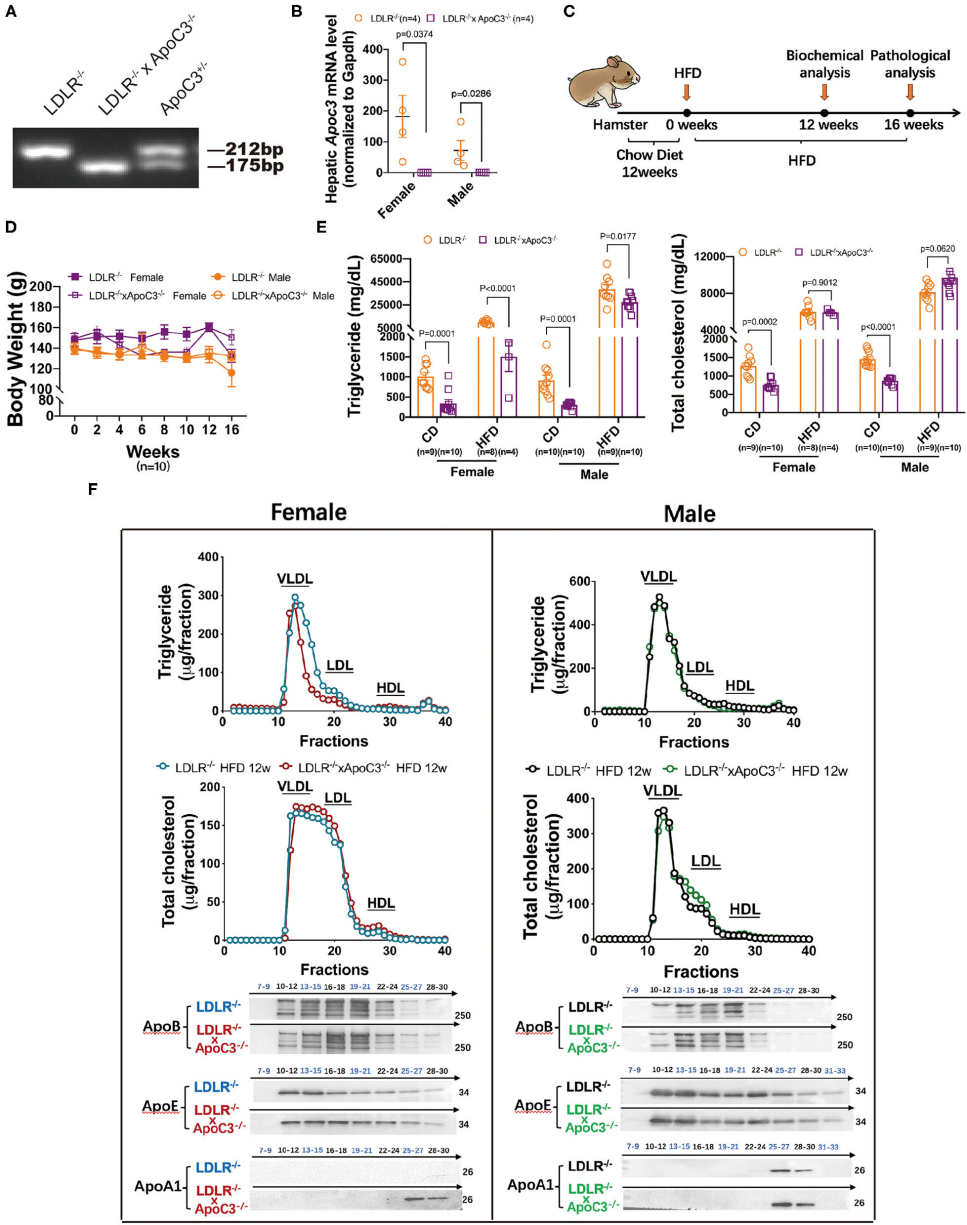
Effects of ApoC3 deficiency on lipid metabolism in LDLR^−/−^ hamsters. **(A)** Generation of DKO hamster model. **(B)** Hepatic *Apoc3* mRNA expression levels were determined in indicated animals on chow at 12 weeks old. **(C)** Timeline of the whole experiments in this study. High-cholesterol/high-fat diet (HCHF) contains 0.5% cholesterol and 20% fat. **(D)** Measurement of body weight during the 16-week experiment. **(E)** Fasting plasma TG and TC levels in the indicated animal on chow or HCHF diet. **(F)** The distribution of TG and cholesterol in pooled plasma samples from the indicated animals after 12-week HCHF diet feeding and representative western blots of ApoB, ApoE, and ApoA1 in eluted fractions. *n* = 4–5/group. Data are expressed as mean ± SEM, analyzed by two-way ANOVA or Student's *t*-test after D'Agostino–Pearson's normality test using Prism 8.0.

To investigate the cholesterol-lowering effect of depleting ApoC3 in hamsters, in this study, we crossed ApoC3-deficient hamsters with a background of LDLR deficiency to generate a DKO hamster model (Figures 1A,B). At the age of 12 weeks, both LDLR^−/−^ hamsters and DKO hamsters with different genders on standard laboratory diet were switched to the HCHF diet for another 16 weeks to study diet-induced obesity, dyslipidemia, and atherogenesis (Figure 1C). We found that ApoC3 deficiency did not accelerate obesity in DKO hamsters when compared to controls (Figure 1D). Before the HCHF diet challenge, both female and male DKO hamsters on standard laboratory diet showed lower levels of TG and TC in plasma (754 mg/dl vs. 1272 mg/dl in females, 868 mg/dl vs. 1453 mg/dl in males). Consistently, lipoprotein distribution analysis by FPLC demonstrated that the contents of TG and cholesterol in VLDL particles were significantly reduced in DKO hamsters compared to LDLR^−/−^ hamsters (Supplementary Figure 1). However, there were no significant differences in plasma TC levels between two genotypes on the HCHF diet for 12 weeks, but only TG level was partially decreased by 85% in female DKO relative to corresponding controls (Figure 1E). Next, to avoid the obstruction of FPLC by very large TG-rich lipoprotein particles caused by HCHF diet application, we removed chylomicrons from our pooled plasma samples and found that only female DKO hamsters consistently showed reduced contents of TG in VLDL fractions but no changes in LDL and HDL fractions (Figure 1F). Additionally, western blots demonstrated that compared to LDLR^−/−^ hamsters, lacking ApoC3 significantly increased ApoA1 levels; however, ApoB and ApoE were not altered in both female and male DKO hamsters (Figure 1F). ApoC3 has been reported to be an inhibitor of LCAT activity in the previous study (11). Therefore, we measured the LCAT activity in our hamster model after HCHF diet feeding. We found that compared to the corresponding LDLR^−/−^ hamsters, female and male DKO hamsters showed an increase in LCAT activity by 1.23 and 1.25 (ratio of fluorescence intensity at 390–470 nm), respectively. These data suggested that targeting ApoC3 could differentially influence lipid metabolism depending on gender and dietary intervention.

### ApoC3 Deficiency Aggravates HCHF Diet-Induced Atherosclerosis in LDLR^–/–^ Hamsters but Protects Against Fatty Liver Only in Female LDLR^–/–^ Hamsters

**Figure 2:**
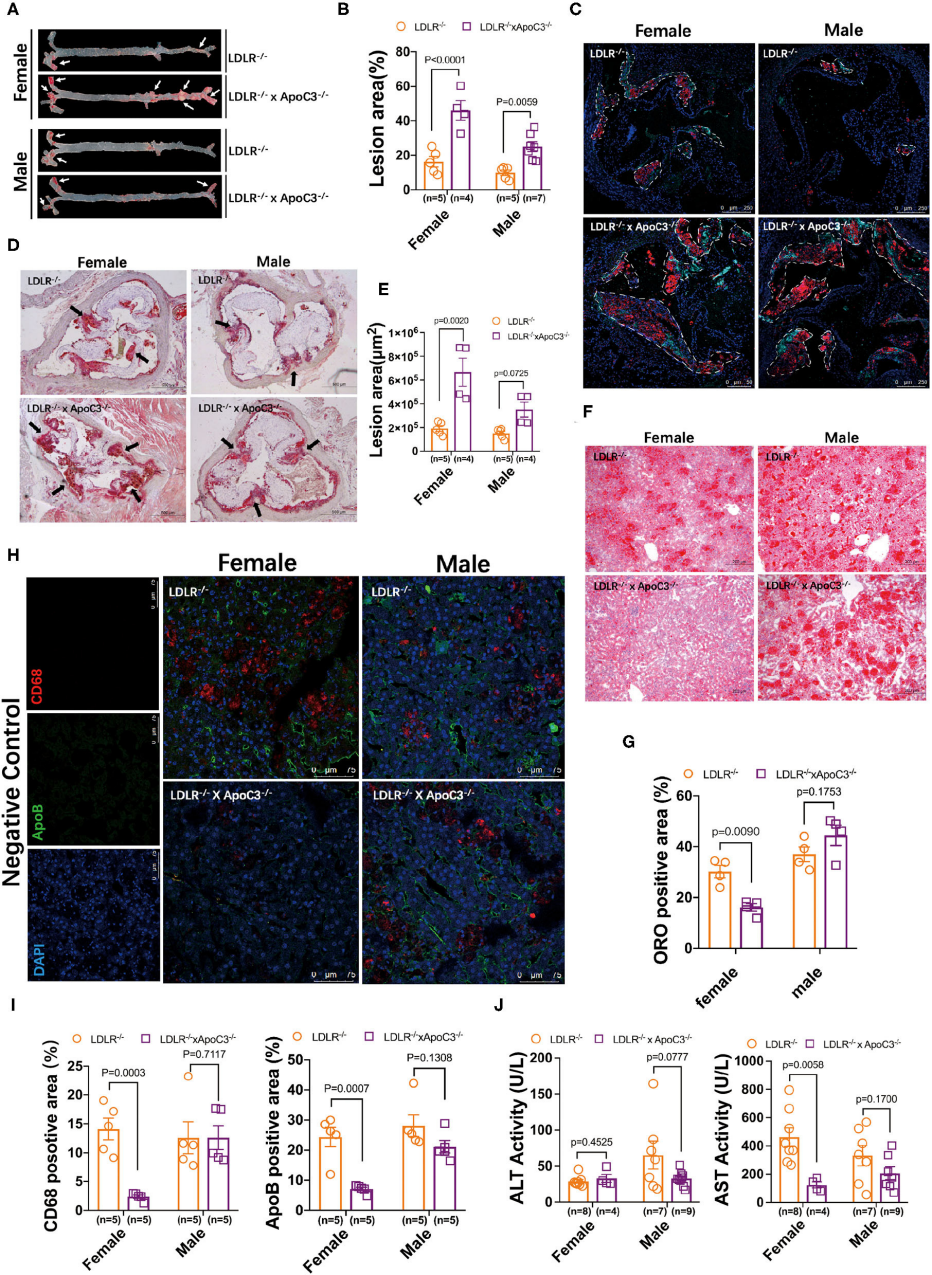
Morphological analysis of atherosclerosis and fatty liver in LDLR^−/−^ and DKO hamsters on HCHF diet. **(A)** Representative en-face images of the whole aorta. Arrows indicate positive staining. **(B)** Quantification of the lesion sizes in the whole aorta. **(C)** Analysis of the components in atherosclerotic plaques by immunofluorescence in HCHF diet-fed animals. Representative staining of ApoB and CD68 in the atherosclerotic lesions of the aortic root. The areas enclosed by white-dotted lines show the plaques in the aortic root. **(D)** Representative ORO staining of the aortic root. Black arrows indicate ORO-positive staining. **(E)** Quantification of the lesion areas in the aortic root. **(F)** Representative images of ORO-stained cryosections from liver samples. *n*=4/group. **(G)** Quantification of the ORO positive areas in the aortic root from **(F)**. **(H)** Immunofluorescence analysis of macrophage infiltration (CD68) in the liver. *n* = 4/group. **(I)** Quantification of the positive sizes in the liver. **(J)** Fasting plasma ALT and AST levels after 12-week HCHF diet feeding. Data are expressed as mean ± SEM, analyzed by two-way ANOVA or Student's *t*-test after D'Agostino–Pearson's normality test using Prism 8.0.

Previously, our group found that ApoC3 deficiency generated favorable lipoprotein profiles, then protecting against diet-induced atherosclerosis in hamster (9); however, whether ApoC3 inhibition still executed long-term beneficial function in atherosclerosis, a major event in FH with hypercholesterolemia due to lacking LDLR. Surprisingly, in HCHF-fed animals, we found that both female and male DKO hamsters developed more atherosclerotic plaques in the whole aorta when compared to the corresponding LDLR^−/−^ hamsters (46.04 vs. 16.25% in females; 27.14 vs. 10.64% in males; Figures 2A,B). Consistent with the results of atherosclerotic plaques observed in the whole aorta, the areas of atherosclerotic lesions in aortic roots were also significantly increased in DKO hamsters relative to control animals (6.7×10^5^ μm vs. 1.9×10^5^ μm in females; 3.5×10^5^ μm vs. 1.7×10^5^ μm in males; Figures 2C,D). To better understand the composition of atherosclerotic lesions, we performed immunofluorescence staining to analyze ApoB and CD68, two key markers of lipid-loaded macrophages accumulated in atherosclerotic lesions (Figure 2E). Our data showed that more ApoB and CD68 were discovered in the lesions of DKO hamsters regardless of gender, demonstrating that loss of ApoC3 paradoxically accelerated atherosclerotic development in both female and male DKO hamsters in a lipid-independent manner, accompanied by abnormal infiltration of lipid-loaded macrophages. Recently, the approach trial using volanesorsen showed thrombocytopenia in patients of familial chylomicronemia syndrome (FCS), who received ASO-mediated inhibition of ApoC3. Thus, it was rational for us to determine PLT indices in our animal model, which have been reported to play an essential role in atherosclerosis. We measured the number of PLT, MPV, PDW, and PCT and found that platelet indices were abnormal to some extent in both female and male DKO hamsters when compared to the corresponding LDLR^−/−^ hamsters, implying that abnormal platelet function could be a potential contributor to atherosclerosis in our study.

Since the relationship between ApoC3 and fatty liver is still elusive in different mouse models, we also studied the hepatic morphology in our hamster model. ORO staining revealed that hepatic lipid accumulation was markedly increased in female DKO hamsters compared to LDLR^−/−^ hamsters with the same gender, but no obvious difference was observed in male animals (Figures 2F,G). In agreement with the findings of lipid contents stained by ORO, immunoflurorescent detection of ApoB and CD68 in the liver showed that hepatic ApoB and CD68 levels were significantly reduced in only female DKO hamsters (Figures 2H,I), accompanied by a reduced level of aspartate aminotransferase (AST), one of the predictors of liver injury (Figure 2J).

## Discussion

Although ApoC3 inhibition has been applied for the treatment of HTG in different clinical trials, however, unlike ANGPTL3, an inhibitory regulator of LPL activity was recently accepted as a potential therapeutic target for both HTG and hypercholesterolemia, whether targeting ApoC3 has beneficial effects on refractory hypercholesterolemia and consequential outcome of atherogenesis in the context of FH due to dysfunctional LDLR is still elusive. In this study, we developed a DKO hamster model lacking both ApoC3 and LDLR. Our data showed that depleting ApoC3 by CRISPR/Cas9 gene editing significantly reduced circulating TG and TC levels in LDLR^−/−^ hamsters on standard laboratory diet, but only lowered plasma TG concentration in HCHF diet-fed female LDLR^−/−^ hamsters without affecting severe refractory hypercholesterolemia. Unexpectedly, diet-induced atherosclerosis was aggravated in DKO hamsters independent of gender, whereas female DKO hamsters were protected from fatty liver. Our findings indicate that complete loss of ApoC3 plays favorable effects on HTG and fatty liver in the setting of severe refractory hypercholesterolemia only in female LDLR^−/−^ hamsters, but paradoxically elicits atherosclerotic development regardless of gender.

A growing body of evidence demonstrates that increased plasma ApoC3 levels are positively associated with elevated circulating TG concentration and increased incidence of CVD in independent population-based studies (12). Consistently, transgenic mice overexpressing human ApoC3 also show HTG and accelerated diet-induced atherosclerotic development in atherosclerosis-prone LDLR^−/−^ mice (13). However, whether reduced plasma ApoC3 levels are cardioprotective is still under debate because the results from both human and experimental animal studies are contradictory. Human knockouts of ApoC3 study revealed that ApoC3 deficiency decreased plasma TG and cholesterol levels, and increased HDL-C levels, suggesting an atheroprotective lipoprotein profile (14). But recent Mendelian randomization studies led by Goyal et al. discovered that only one rare loss-of-function variant (rs138326449) was correlated with reduced plasma TG levels and lower risk of cardiovascular heart disease (CHD) in Europeans but not in Asian Indians. Of note, another five common loss-of-function variants of ApoC3, including rs373975305 (IVS1-2G-A), rs76353203 (R19X), rs138326449 (IVS2+1G-A), rs147210663 (A43T), and rs140621530 (IVS3+1G-T), did not show cardioprotective property even though they differentially influenced plasma TG levels in Asian Indians (15). In addition, Li et al. found that deletion of ApoC3 from LDLR^−/−^ mice has no impact on lipid metabolism and atherogenesis, indicating that targeting ApoC3 to treat hyperlipidemia and CVD needs to be further validated.

In our study, our results showed that ApoC3 deficiency improved combined hyperlipidemia with elevated TG and cholesterol in LDLR^−/−^ hamsters on a standard laboratory diet, which was consistent with the lipid-lowering effect observed in HCHF diet-fed wild-type hamsters (10). Unfortunately, upon HCHF diet feeding, severe refractory hypercholesterolemia was not ameliorated in DKO hamsters, whereas partial reduction in TG was observed in only female DKO hamsters. These findings demonstrated that loss of ApoC3 still attenuated severe HTG in the setting of severe refractory hypercholesterolemia in female FH hamster model after lipid-rich diet challenge, indicating that sex hormone could potentially contribute to lowering TG. Interestingly, in patients with FCS caused by loss-of-function mutation P207L/P207L in *LPL* gene, TG reduction was more prominent in females than in male patients after receiving ISIS304801, an inhibitor of *Apoc3* messenger RNA (mRNA) (16). However, the molecular mechanism by which sex hormones, including estrogen and testosterone, regulate TG metabolism through ApoC3/LPL pathways has not been fully understood yet. It will be tempting to perform ovariectomy in female hamsters or castration in male hamsters to solve this unanswered issue in future studies.

Although ApoC3 deficiency did not alter severe refractory cholesterol levels in LDLR^−/−^ hamster on HCHF diet, we observed an obvious increase in plasma ApoA1 concentration in HDL fractions separated by FPLC in both female and male animals. We speculated that the elevated ApoA1 levels could be attributed to increased LCAT activity because a previous study reported that LCAT activity was significantly reduced in human ApoC3 transgenic mice, suggesting that ApoC3 was an inhibitor of LCAT activity (11). In accordance with the results gained from the mice overexpressing human ApoC3, we found that LCAT activity was increased in DKO hamsters when compared to LDLR^−/−^ hamsters after HCHF diet feeding. However, although ApoA1 levels were elevated in LDLR^−/−^ hamsters lacking ApoC3, TC and HDL-C levels did not change, which was similar to the observations that overexpression of LCAT in LDLR^−/−^; ob/ob mice only increased LCAT activity by 64%, but did not affect total lipid levels in plasma (17). Thus, we speculated that a 24% increase in LCAT activity in our FH model with ApoC3 deficiency was sufficient to elevate ApoA1 levels but could not influence plasma cholesterol levels, including HDL-C.

Surprisingly, contrary to what was expected from TG-lowering effect of ApoC3 deficiency in the context of severe refractory hypercholesterolemia, our findings showed that DKO hamsters displayed accelerated atherosclerotic plaques with more lipid-load macrophage accumulation in the lesions, which was independent of gender. This paradoxical result raised a possibility that ApoC3 deficiency might enhance the uptake of lipid-rich lipoprotein particles at the surface of the local vascular wall through LPL activity dependent or independent manner because the LPL-mediated catabolism of ApoC3-containing lipoproteins was lower than ApoC3-deficient lipoproteins (16). Adenovirus overexpressing active or non-active LPL in the endothelial-intact artery caused lipid deposition in HTG mice with LPL deficiency and hypercholesterolemic mice lacking ApoE (18), which did not affect plasma lipid levels, suggesting that endothelial-associated LPL was proatherosclerotic *in vivo*. Moreover, another independent study led by Ferna'ndez-Hernando from Yale reported that lack of hematopoietic cell-derived ANGPTL4, one of the inhibitors of LPL, also promoted atherosclerosis by regulating monocyte expansion in LDLR^−/−^ mice (19). Collectively, these data demonstrated that suppressing the inhibitory effect of LPL inhibitors at the vascular wall might trigger atherosclerotic development. Importantly, thrombocytopenia has been reported in FCS patients with volanesorsen treatment; however, this study showed that depleting ApoC3 by CRIPSR/Cas9 resulted in abnormal platelet indices without thrombocytopenia, indicating that genetically targeting ApoC3 could not significantly cause decreased platelet number; however, the abnormal platelet function due to ApoC3 deletion would be considered as another potential contributor to atherosclerosis in our study. It should be noted that in this study, we only investigated atherosclerosis under the HCHF diet condition, which predisposed LDLR^−/−^ hamsters and DKO hamsters to severe refractory hypercholesterolemia that could not be improved by the absence of ApoC3; however, ApoC3 deficiency generates a favorable plasma lipoprotein profile by reducing both TG and cholesterol levels in LDLR^−/−^ hamsters, suggesting that currently, we cannot exclude the long-term beneficial effect of ApoC3 inhibition on spontaneous atherosclerosis in LDLR^−/−^ hamsters on standard laboratory diet, which should be validated in future.

Non-alcoholic fatty liver disease (NAFLD) is the consequence of hyperlipidemia in humans and experimental animals. Our data clearly showed that ApoC3 inhibition obviously reduced lipid contents in female LDLR^−/−^ hamsters after HCHF diet feeding, which was consistent with plasma lipid levels, suggesting that the TG-lowering effect of ApoC3 inhibition effectively ameliorated fatty liver by reducing macrophage infiltration, then protecting from liver damage. Again, although we still do not understand why TG was reduced only in female DKO, our data suggest the need for future investigation of the effect of sex hormones in our model.

In conclusion, we have demonstrated that targeting ApoC3 could reduce TG levels and ameliorate fatty liver only in female FH hamsters with severe refractory hypercholesterolemia after HCHF diet feeding, but paradoxically accelerated diet-induced atherosclerotic development in dependent on gender. Our unexpected findings provide new insight into the possibility that targeting ApoC3 will be a potential therapeutic approach for the treatment of FH with severe refractory hypercholesterolemia and atherosclerosis.

## Data Availability Statement

The datasets presented in this study can be found in online repositories. The names of the repository/repositories and accession number(s) can be found in the article/Supplementary Material.

## Ethics Statement

The animal study was reviewed and approved by the Animal Care Committee, Peking University Health Science Center (LA2015-012).

## Author Contributions

XX conceived and designed the study. YX, JG, LZ, GM, PL, and WZ performed the experiments. YX, JG and XX wrote the original manuscript. YX, JG, LL, XH, YW, WH, and XX interpreted the data. YW, GL, and XX acquired the funding. GL and XX supervised the study. MG and XX reviewed and edited the manuscript. All authors approved the final manuscript.

## Funding

This study was supported by the National Natural Science Foundation of China (NSFC) 31520103909 and 91739105 to GL, 81770449 to YW, and 82070460 to XX. GL is a fellow at the Collaborative Innovation Center for Cardiovascular Disease Translational Medicine, Nanjing Medical University.

## Conflict of Interest

The authors declare that the research was conducted in the absence of any commercial or financial relationships that could be construed as a potential conflict of interest.

## Publisher's Note

All claims expressed in this article are solely those of the authors and do not necessarily represent those of their affiliated organizations, or those of the publisher, the editors and the reviewers. Any product that may be evaluated in this article, or claim that may be made by its manufacturer, is not guaranteed or endorsed by the publisher.

## Abbreviations

ApoA1: apolipoprotein A1
ApoB: apolipoprotein B
ApoE: apolipoprotein E
ApoC3: apolipoprotein C3
HDL: high-density lipoprotein
LDL: low-density lipoprotein
LDLR: low-density lipoprotein receptor
VLDL: very low-density lipoprotein
FH: familial hypercholesterolemia
HCHF: high cholesterol/high fat
LPL: lipoprotein lipase
TG: triglyceride
HTG: hypertriglyceridemia
ASCVD: atherosclerotic cardiovascular disease.
