# Supplementary material for: Targeting ApoC3 Paradoxically Aggravates Atherosclerosis in Hamsters With Severe Refractory Hypercholesterolemia
Source: Front Cardiovasc Med. 2022 Feb 2;9:840358. doi: 10.3389/fcvm.2022.840358 (PMC8847384; doi:10.3389/fcvm.2022.840358)
Supplement: Supplementary file 1 [file Data_Sheet_1.docx]

**SUPPLEMENTAL MATERIALS**

**Targeting ApoC3 paradoxically aggravates atherosclerosis in hamsters with severe refractory hypercholesterolemia**

Yitong Xu^1*^, Jiabao Guo^2*^, Ling Zhang^2*^, Guolin Miao^2^, Pingping Lai^2^, Wenxi Zhang^2^, Lili Liu^3^, Xinlin Hou^3^, Yuhui Wang^2^, Wei Huang^2^, George Liu^2^, Mingming Gao^1#^, Xunde Xian^2,4#^†

1 Laboratory of Lipid Metabolism, Institute of Basic Medicine, Hebei Medical University, Shijiazhuang, China;

2 Institute of Cardiovascular Sciences and Key Laboratory of Molecular Cardiovascular Sciences, Ministry of Education, School of Basic Medical Sciences, Peking University, Beijing, China;

3 Department of Pediatrics, Peking University First Hospital, Beijing, China;

4 Beijing Key Laboratory of Cardiovascular Receptors Research, Beijing 100191, China.

***Running title: ApoC3 and atherosclerosis in LDLR^-/-^ hamsters***

*Equal contribution

^#^Correspondence should be addressed to Dr. Xunde Xian PhD (xianxunde@bjmu.edu.cn) or Dr. Mingming Gao PhD (g.m0515@163.com).

†Lead contact

**Major Resources Table**

In order to allow validation and replication of experiments, all essential research materials listed in the Methods should be included in the Major Resources Table below. Authors are encouraged to use public repositories for protocols, data, code, and other materials and provide persistent identifiers and/or links to repositories when available. Authors may add or delete rows as needed.

**Animals (in vivo studies)**

| **Species** | **Vendor or Source** | **Background Strain** | **Sex** |
| --- | --- | --- | --- |
| Hamster | Beijing Vital River Laboratory Animal Technology Co | Syrian golden hamster, LDLR knockout | Female  Male |
| Hamster | Beijing Vital River Laboratory Animal Technology Co | Syrian golden hamster,  LDLR and ApoC3 double knockout | Female  Male |

**Genetically Modified Animals**

|  | **Species** | **Vendor or Source** | **Background Strain** | **Other Information** |
| --- | --- | --- | --- | --- |
| **Parent - Male** | Hamster | Beijing Vital River Laboratory Animal Technology Co | Syrian golden hamster, LDLR knockout | Gene modified animals were generated by CRISPR/CAS9 technology |
| **Parent - Female** | Hamster | Beijing Vital River Laboratory Animal Technology Co | Syrian golden hamster, LDLR knockout | Gene modified animals were generated by CRISPR/CAS9 technology |
| **Parent - Male** | Hamster | Beijing Vital River Laboratory Animal Technology Co | Syrian golden hamster, ApoC3 knockout | Gene modified animals were generated by CRISPR/CAS9 technology |
| **Parent - Female** | Hamster | Beijing Vital River Laboratory Animal Technology Co | Syrian golden hamster, ApoC3 knockout | Gene modified animals were generated by CRISPR/CAS9 technology |

**Antibodies**

| **Target antigen** | **Vendor or Source** | **Catalog #** | **Working concentration** | **Lot # (preferred but not required)** | **Persistent ID / URL** |
| --- | --- | --- | --- | --- | --- |
| ApoB | Millipore | 178467 | 1:5000/1:100 |  |  |
| ApoE | Millipore | 178479 | 1:5000 |  |  |
| ApoA1 | SCB | sc-30089 | 1:1000 |  |  |
| CD68 | BOSTER | BA3638 | 1:100 |  |  |

**Other**

| **Description** | **Source / Repository** | **Persistent ID / URL** |
| --- | --- | --- |
| AAV-hLCAT ;AAV-GFP | Beijing FivePlus Molecular Medicine Institute |  |
| TG kit | Zhongsheng Beikong, Beijing |  |
| TC kit | Zhongsheng Beikong, Beijing |  |
| oil red O | Sigma-Aldrich |  |
| LCAT Activity kit | Sigma-Aldrich |  |
| High cholesterol high fat diet | Beijing Botai Hongda Biotechnology Co. LTD |  |
| Standard laboratory diet | Beijing Keao Xieli Feed Co.,Ltd |  |

**Supplemental figures and figure legends**

**
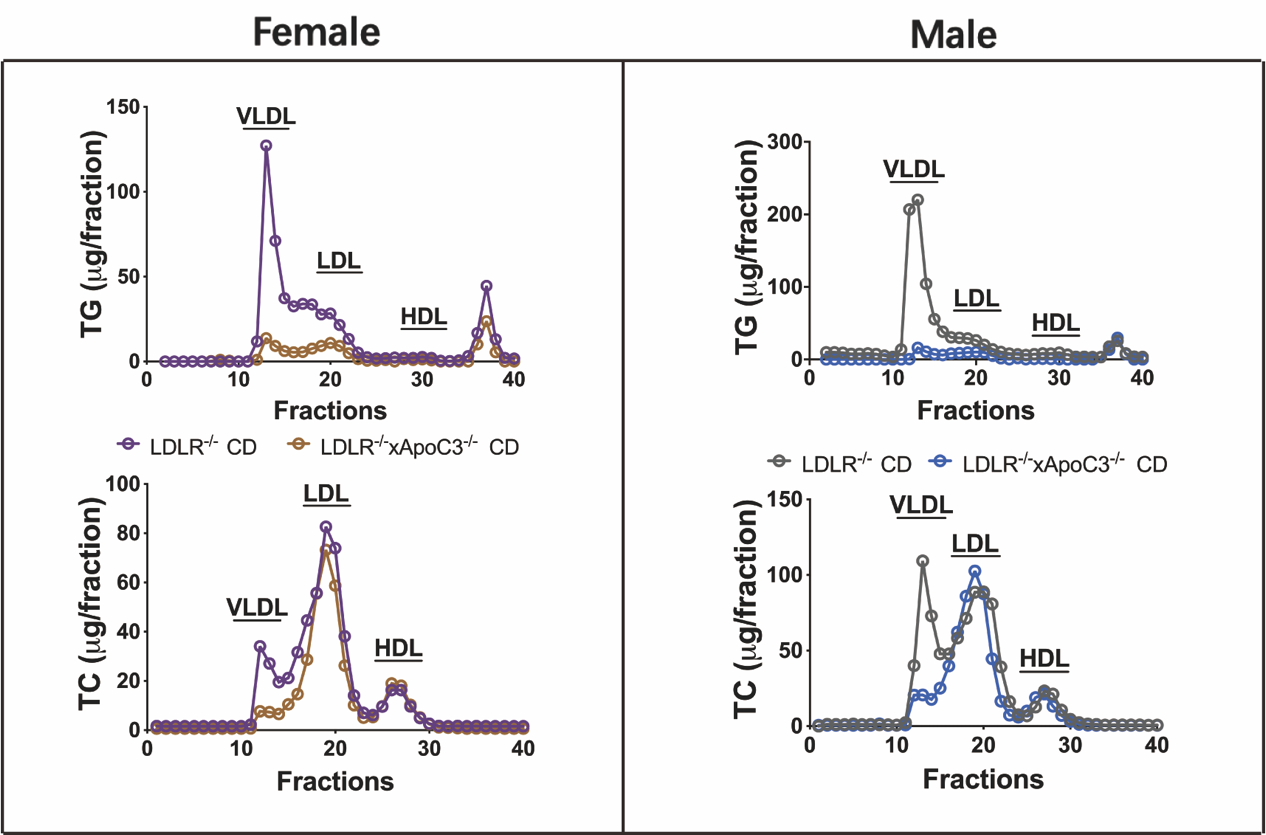
**

**Supplemental Figure I. Analysis of lipoprotein distribution in standard laboratory diet-fed LDLR^-/-^ and LDLR^-/-^XApoC3^-/-^ hamsters at 12-week old age.** The distribution of triglyceride and cholesterol in pooled plasma samples were measured from the indicated animals on standard laboratory diet (n=4-5/group).

**
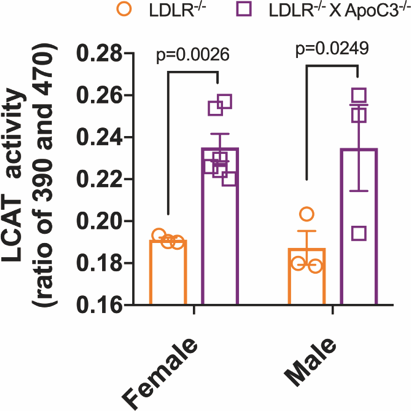
**

**Supplemental Figure II.** **Measurement of LCAT activity.** LCAT activity was determined in LDLR^-/-^ and LDLR^-/-^ XApoC3^-/-^ hamsters on HCHF diet for 12 weeks (n=3-6/group). Data are expressed as mean ± SEM, analyzed by 2-way ANOVA or Student t-test after D’Agostino-Pearson normality test using Prism 8.0.

**
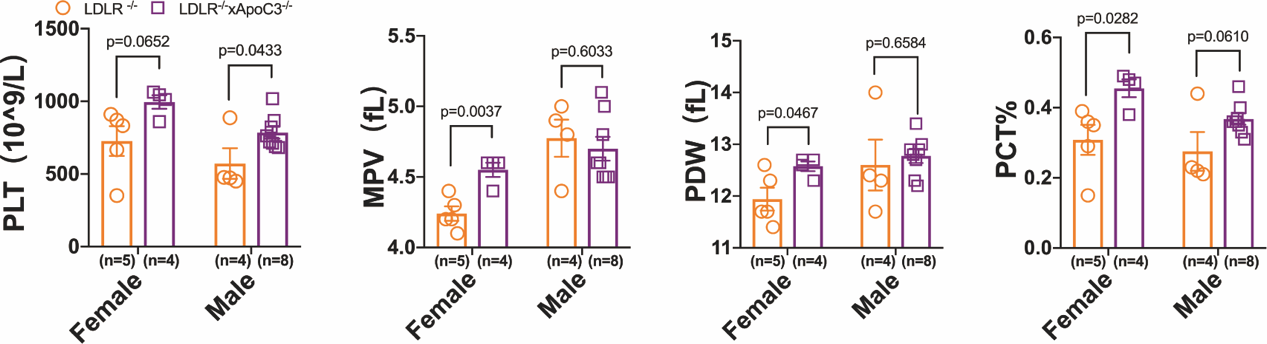
**

**Supplemental Figure III.** Platelet indices measurement. Platelet indices were measured in LDLR^-/-^ and LDLR^-/-^ XApoC3^-/-^ hamsters on HCHF diet for 12 weeks. Data are expressed as mean ± SEM, analyzed by 2-way ANOVA or Student t-test after D’Agostino-Pearson normality test using Prism 8.0.
